# Genetic Improvement of *Camelina sativa* (L.) Crantz: Opportunities and Challenges

**DOI:** 10.3390/plants12030570

**Published:** 2023-01-27

**Authors:** Martina Ghidoli, Elena Ponzoni, Fabrizio Araniti, Daniela Miglio, Roberto Pilu

**Affiliations:** 1 Department of Agricultural and Environmental Sciences—Production, Landscape, Agroenergy, Università degli Studi di Milano, Via G. Celoria 2, 20133 Milan, Italy; 2 Institute of Agricultural Biology and Biotechnology, Consiglio Nazionale delle Ricerche, Via E. Bassini 15, 20133 Milan, Italy; 3 Laboratory for Mother and Child Health, Department of Public Health, Istituto di Ricerche Farmacologiche Mario Negri IRCCS, 20133 Milan, Italy

**Keywords:** *Camelina sativa*, oilseed crops, breeding, GMO, genome editing

## Abstract

In recent years, a renewed interest in novel crops has been developing due to the environmental issues associated with the sustainability of agricultural practices. In particular, a cover crop, *Camelina sativa* (L.) Crantz, belonging to the Brassicaceae family, is attracting the scientific community’s interest for several desirable features. It is related to the model species *Arabidopsis thaliana*, and its oil extracted from the seeds can be used either for food and feed, or for industrial uses such as biofuel production. From an agronomic point of view, it can grow in marginal lands with little or no inputs, and is practically resistant to the most important pathogens of *Brassicaceae*. Although cultivated in the past, particularly in northern Europe and Italy, in the last century, it was abandoned. For this reason, little breeding work has been conducted to improve this plant, also because of the low genetic variability present in this hexaploid species. In this review, we summarize the main works on this crop, focused on genetic improvement with three main objectives: yield, seed oil content and quality, and reduction in glucosinolates content in the seed, which are the main anti-nutritional substances present in camelina. We also report the latest advances in utilising classical plant breeding, transgenic approaches, and CRISPR-Cas9 genome-editing.

## 1. Introduction

*Camelina sativa* (L.) Crantz, also called gold-of-pleasure, false flax, or linseed dodder, is an oilseed crop belonging to the tribe Camelineae of the mustard family (*Brassicaceae*) [1,2,3].

**Figure 1:**
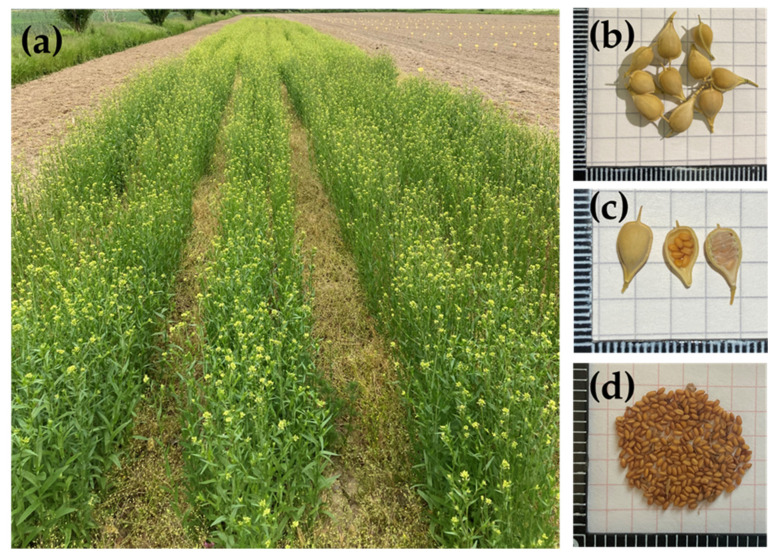
*Camelina sativa* crop. (**a**) Flowering plants; (**b**) silique; (**c**) opened silique; (**d**) seeds.

Plants are erect and typically reach heights between 30 and 90 cm. Rosette leaves are not lobed and are withered by the time of flowering. The stems are branched, woody when mature, and can be sparsely hairy. The leaves alternate on the stem and are lanceolate with a length of 2–8 cm and a width of 2–10 mm. Inflorescences are racemes with small flowers in terminal clusters. The flowers are pale yellow with four spatulate petals. The siliques are 7 to 9 mm long, leathery, smooth, and usually contain 5–15 golden brown seeds. Seeds are small, generally 2 to 3 mm long, brown in colour, rough, and have a rippled surface (Figure 1).

The weight of 1,000 seeds is in the range of 0.8 to 2.0 grams. The seeds contain 38 to 43% oil, and 27% to 32% protein. Camelina reproduces through seed and is primarily a self-pollinating species [4,5].

The possible centre of origin is located between Ukraine and Russia. The genetic diversity hotspot was identified in this region [6]. The distribution of camelina extends from Europe to southwestern Asia, and it was introduced in America and Canada as a contaminant of flax, hence the name false flax. *C. sativa* is a very ancient crop plant, and archaeological evidence suggests that its cultivation began in the Neolithic age in south-eastern Europe and during the Iron age, it was an important crop in most of Europe. In 1950 in Denmark, a mummified human body datable to this age was found from whose remains the contents of the last meal were identified: barley, flax, oats, and camelina. During the Roman Empire, the oil extracted from the seeds of this plant was used for lamps, body care, and food. In 600 BC, camelina was cultivated in the Rhine valleys as a monoculture. Its cultivation continued until 1940 throughout France, Belgium, and Russia, where the oil was also used as fuel. Since 1950, this crop has been abandoned and replaced with more profitable crops [2,7,8].

In the current European scenario, the energy crops (based on rotational crops) that provide raw materials, such as sugar, starch, and oils, are becoming affordable and feasible to produce on a large scale. Among these, oil crops currently predominate, covering about 82% of the energy crop area (mainly rapeseed and sunflower). 

In recent years, camelina has been rediscovered, mainly in the United States, Canada, and in Europe. For example, in Italy camelina seems to have great potential thanks to numerous interesting traits [9,10]. Therefore, crops, such as camelina, hemp, flax, crambe, and castor bean (plants that produce highly unsaturated oils great for bio-lubricants), are coming back into favour [2,11,12,13].

Genetic studies of the genome of camelina suggest a polyploid structure, and it is most likely a hexaploid species (genome size of 750 Mbp with 2n = 40 chromosomes) [5]. Camelina seeds contain up to 40% oil, 90% of which is made up of unsaturated fatty acids: 30–40% fraction of α-linolenic acid, 15–25% fraction of linoleic acid, 15% fraction of oleic acid, and around 15% eicosenoic acid [1]. Camelina oil is considered a high-quality edible oil [14]. This crop is interesting because it has several attractive traits, such as a high ability to adapt to marginal soils and low input growing conditions. Moreover, it has a high resistance of the siliques to dehiscence [13,15].

Three main goals should be achieved to enhance the cultivation of *Camelina sativa* L. not only as a cover crop, but to increase production and economic value [16]. The first goal should be yield improvement since camelina spring cultivars’ productivity is not high (1.5–2.5 t/ha) [17]. 

The second is the oil content and quality. Furthermore, the increase in the human and livestock population is leading to a greater need to find new sources of proteins and oils [17].

The third is to reduce glucosinolate content. It is important to know which are the anti-nutritive compounds in camelina seeds to use in nutrition, especially the secondary plant metabolites, such as glucosinolates, sinapine, phytic acid, and condensed tannins [18]. 

In this review, we will show the more promising advancements obtained up to date by using classical plant breeding, transgenic approaches and CRISPR-Cas9 genome editing, and discuss the pros and cons of the different approaches.

## 2. Cultivation

Global climate change is leading to the deterioration of the sustainability of various economic sectors worldwide. In particular, the most affected sector that causes the greatest concern is the agricultural sector, which has been increasingly looking for crops that can be as resilient as possible to this irreversible climatic variability [19,20]. Crop diversification is used to promote better environmental, social, and economic sustainability of agri-food systems, maintaining their production capacity, providing ecosystem services, and promoting the efficient use of resources.

Camelina is a hardy plant that adapts very well to different types of soil and grows best in cool semi-arid climates. Camelina can tolerate drought conditions, although they can negatively impact sensitive growth phases, such as flowering [4,21]. Different works conducted in different countries worldwide on camelina seed yield were reviewed by Berti and co-authors [3]. Reported yields vary greatly depending on the climate, the cultivar used, and soil type. However, the highest seed yields have been registered in Mediterranean climates [2,10,19,22,23].

In the western Prairie provinces of Canada and the North and Central Plains in the USA, camelina may be economically competitive with other alternative oilseeds common to these areas, such as soybean (*Glycine max* (L.), flax (*Linum usitatissimum* L.), rapa canola (*Brassica rapa* L.), juncea canola (*Brassica juncea* L.), yellow mustard (*Sinapis alba* L.), oriental mustard (*Brassica juncea* L.), and Ethiopian mustard (*Brassica carinata* L.) [24]. In the upper Midwest Corn Belt region, camelina cultivation as a standalone crop could not be competitive with corn and soybean, and should be used in winter dual cropping to integrate the corn–soybean systems [25].

Its cultivation is also arousing growing interest in Italy [11,21,26]. In fact, a yield of 1200–3300 kg/ha in the Italian Lombardy Region was reported using seven different spring varieties (Calena, Ligena, Ukrajinskaja, Lindo, Zarja Socialisa, Soledo, and Morgesonne) [11].

In this paper, the agronomic performance over two consecutive years of camelina sown in spring and autumn was evaluated in comparison with rapeseed (*Brassica napus* L.). The result showed, in general, the seed yield is similar to that of the rapeseed control and, on average, between 1340 and 1625 kg/ha. Furthermore, regarding the two sowing seasons, autumn planting allowed a better yield [11].

Camelina can be used in intercropping and rotation systems, especially in drier areas [1,3,19].

Winter genotypes are the best varieties for growing in winter to protect the soil. Concluding, using winter camelina as a cover crop prevents erosion and promotes carbon sequestration in the soil. Moreover, it can also be used to control weeds as it inhibits their growth [27,28].

## 3. Uses and Potential

In recent years, the interest in this plant has increased significantly as an oilseed crop for food, feed, jet fuel, and bio-based products [13,24]. Berti and colleagues reported the great potential of the crop and its numerous uses, particularly the oil properties and composition, which are useful for the purposes reported in Table 1 [3].

Camelina can be used to improve the quality of foods, such as dairy products and meat, and the consumption of its oil has potential benefits for human health [73]. The oil is rich in essential omega-3 fatty acids (e.g., α-linolenic acid) associated with reducing the risks of coronary and inflammatory diseases. High polyunsaturated fatty acids content could reduce blood serum cholesterol levels [74], and improve serum lipid profiles [75] while protecting against cardiovascular risk factors. In folk medicine, camelina oil was used to treat skin wounds and burns [76].

In addition to the various benefits, the high levels of tocopherols and phytosterols with antioxidant activity increase the shelf life and stability of the oil [18,77].

Several studies have demonstrated the usefulness of camelina meal as a component of feed for broilers, cattle, dairy cattle, and fish, such as salmon (Table 1), with the added benefit of increasing the omega-3 content [50,78]. Camelina meal obtained from high-pressure seed crushing, or a pre-press solvent extraction process represents an important output with considerable economic value. 

In animal feed, camelina flour and seeds are considered beneficial in limited quantities [19]. The presence of anti-nutritional compounds limits its use in zootechnical nutrition with a maximum percentage of 10% [79]. However, compared to other brassicas, the sinapine content (sinapines are alkaloids present in the seeds of *Brasssicaceae* that reduce the digestibility of proteins) is lower in the camelina meal. The glucosinolate content is mainly considered to evaluate the palatability, and it can be included with a content of 27–32 mmol/kg glucosinolates (GSLs) [80].

Additionally, considering the glucosinolates, camelina’s accessions with a low content of erucic acid can be selected for animal diets. In the US Department of Agriculture’s (USDA) National Genetic Resources Program collection, erucic acid content varies from 1.8–4.8% in camelina seed meal, and for feed, it is limited to a maximum of <2% [19]. Neupane et al. [19] evaluated the effects of camelina meal on different animals’ diets. Adding camelina flour or seed oil to the diets of dairy cows led to an increase in the MUFA (monounsaturated fatty acid) and PUFA (poly unsaturated fatty acid) content in the milk with a consequent decrease in saturated fatty acids, without altering other parameters, such as the intake of dry matter, milk production, or digestibility.

In sheep diets, the addition of camelina flour increased the total omega-3 content and improved the omega-6/omega-3 ratio in both lamb meat and milk, the oxidative stability of the milk increased, and there was a reduction in atherogenic and thrombogenic indices. In pigs’ diet, the inclusion of camelina seed meal by up to 18% increased the content of α-linolenic acid and reduced the cholesterol content of the meat, thus improving its quality. In addition to the quality of the final product, the animals’ health improved. Camelina meal and oil are excellent substitutes for fish meal and fish oil in fish feed. Several studies have shown an improvement in the total lipid content in salmon (*Salmo salar* L.) and cod (*Gadus morhua* L.) without affecting the sensory quality. The use of camelina in fish feed improved the content without adversely affecting the sensory quality of the fish fillets. Replacing fish oil with camelina oil had no effect on growth performance for most fish. It also tends to increase the omega-3 PUFA content in meat [48,50]. Another work published in 2020 reported the effect of different percentages of camelina cake in laying hens’ diet. Additionally, in this paper the authors showed that the inclusion of up to 20% of camelina cake in the feed did not modify the eggshell quality or the birds’ welfare and health [39].

The great potential of this crop is also being exploited to obtain a sustainable feedstock for its different applications, and to improve dryland agriculture [16].

In addition, the oil was used as a fuel for lamps and in various industrial applications, while the stems were evaluated for their fibre [4,8].

Currently, camelina oil is used as a raw material to produce biofuels, especially for the aviation industry, as it is rich in unsaturated fatty acids, and, consequently, it does not solidify at low temperatures [81]. Recently, in Italy, experiments on the cultivation of this crop for the production of biodiesel and the extraction of pure vegetable oil obtained promising results [9]. The results obtained as “camelina fuels” are encouraging, having successfully surpassed the techno-economic and life cycle analyses (LCA) as a second-generation biofuel [81,82,83].

Camelina also has a high application potential in the chemical industry due to the presence in its oil of a distinctive fatty acid composition. The predominance of polyunsaturated fatty acids means that their use is multiple in the production of biopolymers, bioactive molecules, lubricants, adhesives, varnishes, paints, pharmaceuticals, cosmetics products, and packing materials [17,84]. 

## 4. Antinutritional Compounds: Glucosinolates

Although camelina oil is characterized by a high content of essential fatty acids and is suitable for human consumption, zootechnics, and various industrial applications, it also contains anti-nutritional compounds, such as glucosinolates, synapin, phytic acid, and condensed tannins [85,86,87].

**Figure 2:**
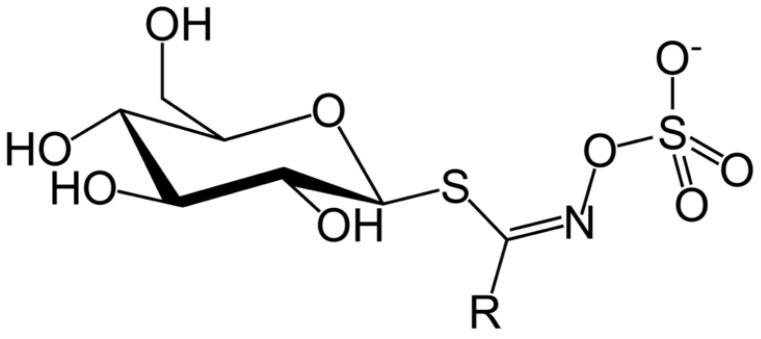
Glucosinolate general structure.

However, the main antinutritional compounds present in *C. sativa* are glucosinolates (GSLs) (Figure 2), secondary metabolites present in the brassicales [88]. These defence compounds are mainly accumulated in high concentrations in seeds, reducing the nutritional value of the protein-rich cake [86]. GSLs tend to form complexes of enzymes and proteins, thus making them indigestible [18].

GSLs are compounds rich in sulphur (β-thioglucoside N-hydroxysulphates with a side chain -R and sulphur linked β-D-glucopyranose) which can be classified by their precursors and the type of modifications of the R group that is mainly elongated by one or more methylene moieties. Glucosinolates’ concentration, composition, and accumulation vary between the stages of development and the plant’s tissues. The GSLs biosynthesis consists of three main steps: condensation with acetyl-CoA, isomerisation, and oxidation-decarboxylation [89]. GSLs, after hydrolysis conducted by myrosinase (thioglucosidase enzyme), are broken down into different catabolites (e.g., thiocyanates, isothiocyanates, nitriles, and epithionitriles) [89,90,91].

In intact plants, GSLs and myrosinases are sequestered in different compartments: the enzyme is associated with the cytoplasmic side of internal membranes, while in extracellular compartments, the GSLs are in vacuoles. In damaged plants, cell decompartmentation allows the enzymatic reaction due to the breakdown of the vacuoles, resulting in the formation of breakdown products. Unlike GSLs, which are inactive molecules, degradation products have different biological effects [89,91,92].

More deeply, glucosinolates can be divided into three main classes: (i) aliphatic, mainly derived from methionine; (ii) aromatic, mainly derived from phenylalanine; and (iii) indolic if their biosynthesis is derived from tyrosine or tryptophane [93].

Simplifying the biochemical process involved in glucosinolates’ biosynthesis, it can be summarised that their formation starts with the insertion of methylene groups in the side chains of aliphatic and aromatic amino acids. Subsequently, the elongated amino acid moiety, through metabolic processes, is reconfigured, giving the typical glucosinolates’ core structure, which will be further modified in structure through various secondary transformations [93]. 

**Figure 3:**
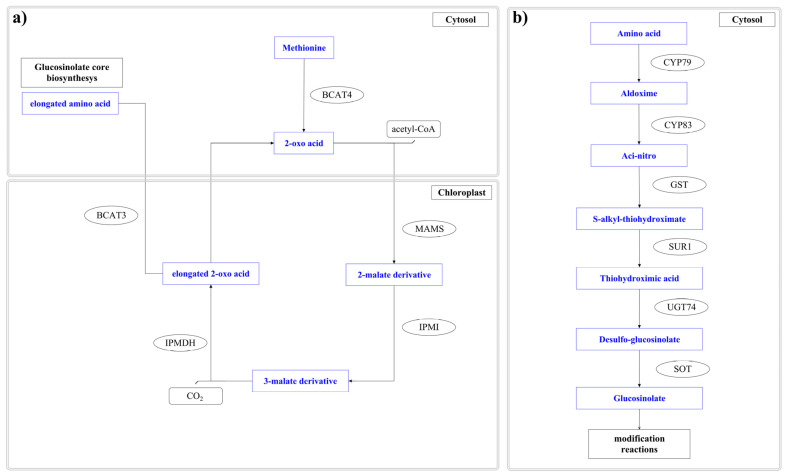
(**a**) Schematic representation of the biochemical processes involved in the aliphatic Glucosinolate Chain Elongation machinery; (**b**) Glucosinolate Core Biosynthesis. Amino acids, including elongated aliphatic methionine-derived molecules, can be converted to aldoximes by members of the CYP79 cytochrome P450 family to start building up the core glucosinolate scaffold. BCAT4—branched-chain aminotransferase 4; MAMS—methylthioalkylmalate synthase; IPMI—isopropylmalate isomerase; IPM-DH—isopropylmalate dehydrogenase; BCAT3—branched-chain aminotransferases-3; CYP79; GST; SUR1; UGT74; SOT. The pathway was built using the open-source software Pathvisio vs. 3.3.0.

The starting point of aliphatic glucosinolates is represented by the building block methionine that, after its amination, catalysed by the enzyme BCAT4 (branched-chain amino acid aminotransferase 4), is converted to the corresponding 2-oxo acid, starting the chain elongation process (Figure 3a) [94,95]. 

This first biosynthetic step happens in the cytosol (where BCAT4 is localized). In contrast, all the other enzymatic activities involved in the elongation process are localized in the chloroplasts [96,97,98,99,100], where the 2-oxo acids formed are transported by the chloroplast-localized bile acid transporter BAT5 [101] (Figure 3a).

In the chloroplasts, the aliphatic chain of the 2-oxo acid is elongated by three enzymes. In particular, the first enzyme involved is methylthioalkylmalate synthase (MAMS), which catalyses the condensation of the acetyl-CoA with the 2-oxo acid forming a 2-malate derivative [96,97,102,103,104], which is isomerized to a 3-malate derivative by the isopropylmalate isomerase (IPMI) [99,105]. Finally, the 3-malate derivative is oxidatively decarboxylated by the isopropylmalate dehydrogenase (IPM-DH) to 2-oxo acid, which elongates the original 2-oxo acid by a methylene group [100,106,107]. The newly formed elongated 2-oxo acid could be transaminated to homomethionine by the plastid-located enzyme branched-chain aminotransferases-3 (BCAT3), or can proceed into the cycle for a new round of chain elongation [108,109] (Figure 3a). It should be highlighted that the overall process generates homomethionine and an array of chain-elongated methionine derivatives.

The newly formed homomethionine is further involved in the core formation of the glucosinolates pathway, which takes place in the cytosol, involving a set of enzymatic reactions shared by the three different glucosinolates’ classes (aliphatic, aromatic, and indolic) (Figure 3b).

Successively, a set of enzymes belonging to the CYP79 gene family, cytochrome P450s, mediate the conversion into aldoximes of the elongated methionine-derived amino acids together with tyrosine, tryptophane, and phenylalanine [110,111,112,113,114,115] (Figure 3b). The previously identified Arabidopsis pathway was recently confirmed by Czerniawski and co-authors, identifying orthologs of Arabidopsis glucosinolate biosynthetic genes in the Camelina published genomes [116].

The side chain structure is the main factor determining the biological activity of glucosinolates [117]. After forming the basic glucosinolates core structure, many changes can occur at the side chain and the glucose moiety. The main secondary modifications that might occur in aliphatic glucosinolates are alkenylations, oxidations, benzoylations, and hydroxylations. In turn, methoxylations and hydroxylations are the main transformations to which indolic glucosinolates are subjected [118,119]. These modifications occur in an organ- and development-specific pattern [120,121]. In camelina, three main aliphatic GSLs were identified: GSL1 (9-methyl-sulfinyl-nonyl-GSL), GSL2 (10-methyl-sulfinyl-decyl-GSL), and GSL3 (11-methyl-sulfinyl-un decyl-GSL) [80,122,123]. Taken together, these major glucosinolates represent about 65 % of the total glucosinolate composition. These long-chain glucosinolates predominate in camelina compared to short-chain glucosinolates in canola [123].

In plants, transport processes for reallocating specialized metabolites with protective activity, such as glucosinolates, is a key process adopted to protect specific tissues with high value for species survival. This was demonstrated in Arabidopsis, where most glucosinolates are translocated to the maturating seeds [121]. Moreover, as previously reported, part of the steps involved in glucosinolate core biosynthesis happens in the cytosol, whereas others occur in the chloroplasts. 

Since glucosinolates are organic anions, they cannot diffuse passively across lipophilic membranes, suggesting that transport proteins mediate their translocation from sources to sinks. Immediately after their production, glucosinolates are distributed to a different extent to the plant’s organs, suggesting a short- and long-distance transport of these molecules.

In the short-distance, the leading transporter performs a pivotal role is the bile acid: sodium symporter family protein 5 (BAT5), which imports 2-oxo acids into the chloroplast for side chain elongation and exports the resulting products into the cytosol for their conversion into glucosinolate [101]. BAT5 is the only member transactivated by the three aliphatic glucosinolate regulators HAG1/MYB28, HAG2/MYB76, and HAG3/MYB29 [101]. Its involvement in the aliphatic GSLs biosynthesis has been demonstrated in Arabidopsis since a BAT5 defective mutant was characterized by a reduction in the aliphatic glucosinolates level [101,124]. Concerning long-distance transport, it has been demonstrated in Arabidopsis that the glucosinolates produced by maternal tissues, such as leaves and siliques, were transported and accumulated into the seeds [125,126,127]. In Arabidopsis, this source/sink aliphatic and indolic glucosinolates translocation network is mediated by transporters GTR1 and GTR2 [high-affinity H^+^/glucosinolate influx symporters, belonging to the ubiquitous peptide transporter (PTR/NRT1) superfamily [128,129]], with a leading role for GTR2 [128,130,131,132]. 

From an economic, productive, and eco-friendly point of view, the possibility of manipulating plants genetically to inhibit the activity of these two transporters could lead to a reduction in glucosinolates accumulation in seeds without altering their biosynthesis, thereby maintaining the inherent defence potential of plants [86,132]. In recent studies on *Brassica juncea,* GTR1 and GTR2 knock-out mutants highlighted changes in plant phenotype. In particular, GTR1 mutants were characterized by slightly reduced glucosinolates in seeds and a significantly lower level in source tissues. However, the GTR2 defective mutant displayed a significant reduction in glucosinolates in seeds and a higher accumulation in leaves and pods [132]. In addition, as a consequence of glucosinolates accumulation in source tissues, GTR2 mutants were characterized by higher resistance to the pest *Spodoptera litura*, suggesting that GTR2 manipulation could ameliorate crop production, either by increasing plant defence ability or by reducing anti-nutritional glucosinolates concentrations in seeds for alimentary purposes [86,132]. Recently, the camelina homologous orthologous genes GTR1 and GTR 2 have also been modified by targeted mutagenesis to lower the GSLs content [133].

Although several studies have shown that GSLs exhibit toxicity to mammals, insects, invertebrates, bacteria, nematodes, and fungi [134], GSLs can also be considered for their benefits. For example, GSLs could reduce and prevent certain diseases in animals and humans, fungicidal and biocidal capacities in plants, and antimicrobial use in the food industry [135,136,137].

## 5. Genetic Resources and Varieties Constitution

To tackle Europe’s dependence on protein supply from other countries, it will be necessary to cultivate new crops rich in proteins. The EU depends on about 80% of protein-rich raw vegetable materials to complete the livestock feed rations usually using corn as a main meal [138]. As reported by the European Commission (EC), on examining many different alternative crops currently available in Europe, they found camelina, containing about 25% of crude protein and 30–40% of oil in the seeds, a promising candidate [10,139]. Camelina for years has been an abandoned crop because of its replacement by more productive oil crops such as rapeseed. Due to this, classical and modern plant breeding techniques have not improved the commercial cultivars available.

Furthermore, several studies suggest that genetic variability among different cultivars is limited, complicating the efforts to develop new promising varieties [6,140,141,142,143,144]. Another genetic aspect complicates the breeder’s work regarding this species: *C. sativa* has an allohexaploid genome (2n = 6x = 40) made by three closely related genomes [145], making very difficult to induce genetic variability through the use of chemical-physical mutagenesis. For these reasons, interspecific crosses have been made, cross-hybridizing camelina with its wild relative *C. macrocarpa*. Nevertheless, the results were not promising for genetic improvement due to lower pollen fertility and seed production [146].

Hence, it would seem that the most promising techniques for developing new varieties are transgenic [5] and genome editing, particularly CRISPR-Cas9 [147]. However, in July 2018, the Court of Justice of the European Union (CJEU) clarified that organisms from new mutagenesis techniques fall within the scope of the EU GMO legislation [148], consequently nullifying the results achieved with this new genetic improvement technique in the EU. Considering all these issues, we should not abandon the intention of improving camelina through classic breeding that will surely complement and support NBT, once the European community comes to terms with chemical-physical mutagenesis. Thus, classical breeding methods for self-pollinated plants, such as camelina, remain valid and useful. For example, there are about 140 commercial varieties registered in the Community Variety Register [149] that could be available for cultivation. Among the major producers of camelina are Canada, USA, Slovenia, Ukraine, China, Finland, Germany, and Austria. For example, Arrow seed [150] is a leading company in the USA. Among the largest easy-to-access collections in the world, we can point out the GRIN [151], where 48 different accessions are collected. Among the agronomic characteristics that breeders have considered are greater seed size, resistance to lodging, greater competitive capacity (e.g., broader leaves), and resistance to herbicides. The increase in disease resistance will have to take into account the resistance to downy mildew (*Peronospora parasitica*), to white rust (*Albugo candida*), and to sclerotinia rot (*Sclerotinia sclerotiorum*) [152]. However, the main interest of scientists/breeders is focused on traits regarding the oil amount and quality. In fact, breeding for modified fatty acid composition via mutagenesis (seeds treated with ethyl methanesulfonate) was performed by Buchsenschutz-Nothdurft and colleagues, obtaining promising lines which showed a higher linolenic acid content (about 30%) in the M3-generation [153]. A paper published more recently by Lolli and colleagues showed that using an improved camelina line with a low level of glucosinolates (obtained by classical breeding using the pedigree method) permits the inclusion of up to 20% of camelina cake in the diet of laying hens without any adverse effect on animal welfare and health, eggshell quality, and production performance [39]. However, the recent advances in the field of genomics will give a great contribution to this species’ genetic improvement by the MAB (Marker-Assisted Breeding). For instance, a recent work published by Li and co-authors evaluated the genetic variation in a worldwide collection of 222 accessions using 161301 SNPs generated by whole-genome resequencing, confirming the low/moderate genetic variability present in this species. However, genome-wide association studies (GWAS) complemented by linkage mapping using RIL population (257 lines) allowed the identification of QTLs associated with seed size, fatty acid composition, seed oil content, flowering time, and plant height [154]. Finally, the results obtained in this work led to the identification of a candidate gene (Cs01g013220) associated with fatty acid composition (FAD2-2 gene, an omega-6 desaturase responsible for the desaturation C18:1 to C18:2) and to flowering time. This latter candidate gene, named *Flowering Locus C* (FLC, Csa08g054450), is one of the most promising traits associated with the cultivation of this species [154]. The results obtained in this work will provide useful molecular tools for future breeding programs.

## 6. Biotechnological Approach

Unlike other crops of the *Brassicaceae* family, camelina has historically not been subjected to extensive breeding, and only a small number of cultivars are available for agricultural purposes, meaning that there is a wide margin to explore its genetic potential. Due to its self-pollinating nature and low genetic variability, different biotechnological strategies are needed to alter the final phenotype. The different biotechnological approaches include gene transformation, ethyl methanesulfonate (EMS)-mutagenesis, genome editing (GE), RNA interference (RNAi), high-throughput EcoTILLING to discover new Single Nucleotide Polymorphisms (SNPs), vectors for the transfer of several genes in one go, protoplast fusion, etc. [5,155,156,157]. 

### 6.1. GMO Technology

*Agrobacterium tumefaciens* can easily transform camelina carrying an engineered plasmid, using a floral dip method, obtaining transgenic seeds in a relatively short period (4–6 weeks) [158] or by in vitro leaf explants cultures [145,159]. Selectable markers could identify transgenic seeds, for example, the red fluorescent protein (DsRed) or mCherry fluorescent protein and identify resistance to specific herbicides or antibiotics (Table 2). 

Camelina transformation efficiency of a single copy insertion of a transgene cassette is 0.8% to 1% [158,178]. In camelina, the transgene(s) expression is generally mediated by constitutive promoters, such as CaMV35S that over-expresses a transgene in most or all tissues at all times. In other cases, the transgene(s) expression is specifically driven by seed-specific expression promoters, often heterologous, similar to the related Arabidopsis FAE1 [171] or SiW6 [162], but also conlinin 1 and 2 from flax [171], seed phaseolin promoter from bean [172], soybean glycinin 1 [58,161,168], and others, as reported in Table 2. Advances in the heterologous introduction of genes into camelina are essentially used to modify or improve a wide range of agronomic and biochemical traits, focusing on manipulating seed oil yield and profile. In fact, the market requests have guided breeding strategies on altered fatty acid (FA) content and composition in vegetable oils, and camelina seed oil is not currently ideal for any single purpose. As already reported, camelina oil and FA content is a polygenic trait influenced mainly by the environment, water availability and temperature during seed filling [179,180]. This is a significant limitation in ensuring good ecogeographical performances of camelina in different locations. Fortunately, over 90% of the *Arabidopsis* genes involved in lipid metabolism were also present in the camelina genome [145]. Many efforts were made to show how lipid biosynthesis in camelina seeds can be redesigned to enable the high accumulation of the target oils. Manipulation of medium-chain FA content increased oleic acid, or the synthesis of unusual lipids, such as the production of omega-7 unsaturated FA, or enhancing the functionality of acetyl-TAGs (triacylglycerols), which are some examples in camelina for industrial purposes, well reviewed by Bansal and Durett [181], Murphy [182] and Sainger et al. [5]. Technological strategies used in this regard include the introduction of very complex constructs containing information for a new pathway. For example, overexpressing multiple heterologous genes from various species has obtained a camelina oil rich in omega-3 long-chain polyunsaturated fatty acids (LC-PUFAs) with high levels of EPA and/or DHA as an alternative to oil fish [49]. An interesting approach to modify the FA composition is using RNAi and antisense approaches, as reported in Table 2. For example, Nguyen et al. [168] obtained lines with high oleic acid up to 50% using a double RNAi knock-down mutant targeting fatty acid desaturase 2 (FAD2) and fatty acid elongase 1 (FAE1). With the same approach, they could also generate RNAi lines deficient in napins (2S albumins) in the seed. Numerous scientific publications reporting on the improvement of camelina’s fatty acid metabolism have shown that there is a positive correlation between the change in fatty acid profile and seed size and protein content [167,174], which are other important traits to improve (Table 2).

An example is described by Duan et al. [166], who heterologously transformed camelina with a chimeric myosin XI-2 gene of *Arabidopsis*. The transgenic plants showed enhanced main stem elongation, and an early flowering and seed set, indicating that the transgene can improve plant growth, total seed number, and yield. Increased protein content in the seed and a consequent seed size rise could also be obtained by starch biosynthesis suppression [169]. The significant number of publications on the genetic improvement of camelina described here is certainly not complete, but it represents an idea of the widespread use of this plant as a model crop. 

To date, the European Union’s (EU) uses the precautionary principle, and research on GMO crops can be conducted only under confined conditions and demands pre-market authorization for any GMO to enter the market, besides post-market environmental monitoring. The European Food Safety Authority (EFSA) and the Member States author[s?] require a risk assessment. The EU’s need for vegetable oil is increasing, and transgenic camelina could cover this demand since it is particularly attractive as an industrial seed oil crop. It should be considered that camelina has a limited ability to outcross to other plant species and non-GM camelina, as demonstrated in greenhouse conditions [182] and in the open field [183]. An opening in this direction was made in 2014 by the United Kingdom government (the Department for the Environment, Food and Rural Affairs, and DEFRA), which allowed the first field trials with transgenic camelina plants expressing high levels of long-chain n-3 fatty acids DHA and EPA by Rothamsted Research with an extension period for another five years of trials from 2019 to 2024 [184].

### 6.2. GE Technology

Genome editing (GE) techniques offer several advantages over the previously described conventional or biotechnological breeding processes, in which multiple genes can be targeted simultaneously without any linkage drag. The Clustered Regularly Interspaced Short Palindromic Repeats (CRISPR)/Cas-based RNA-guided DNA endonuclease system is the most versatile GE tool, with unprecedented ease, accuracy, and high efficiency compared to other editing technologies based on the use of engineered nucleases, such as TALENs (transcription activator-like effector nucleases) or ZFNs (zinc finger nucleases). CRISPR/Cas9 can also be particularly effective as a time-reducing approach. The Cas9 protein, when directed to multiple loci by expressing different sgRNAs can induce simultaneous editing in different parts of the genome, as has already been demonstrated in more than 90 species among food, industrial, or ornamental crops [185]. Notably, the mutations can be stably inherited, and the process appears specific as other genes are not mutated. As for other transformation systems, the receptive genotype, plasmid construct features, and the number of specific target sgRNAs may need to be considered to achieve the desired effort. In camelina, Suneson is the most utilised cultivar in GE studies, and a constitutive promoter, such as CaMV35S or the Egg cell promoter, expressed mainly during seed development, is favoured for Cas9 expression (Table 3).

Camelina is also a good example to demonstrate the power of the CRISPR/Cas9 technique compared to conventional and mutagenesis breeding, where it is tough to modify multiple copies of a gene in different positions on different chromosomes. In fact, despite the complexity of the allohexaploid genome, the three subgenomes are highly undifferentiated in camelina. Although most genes have six copies (three homeologs), these copies can often have high sequence identity [145]. Camelina also exhibits diploid inheritance [192], simplifying the progression of single-copy mutated lines to homozygous status. For these reasons, in this crop, the strategy of designing one or more sgRNAs targeting a conserved region for all three homeologous genes selected, located on three different chromosomes, is the most popular (Table 3). SNPs target sequences, on the other hand, help to determine whether all genes have been efficiently targeted and mutated by the Cas9/sgRNA complex in a single generation [157,186], or allow for assessing the effect of different combinations of modified target alleles in subsequent generations [190]. Furthermore, the presence of many different types of insertions and deletions (in-del mutation) at the same sgRNA target site [188,189], or a different percentage of edited mutants in each homeolog can occur [186,191]. Indeed, the number (1, 2, and 3) of the target homeologs that have been edited in such a complex genome can provide some advantages since a sgRNA could be designed for only one or two of the three subgenomes [133] depending on the final purpose [189]. The consequence of performing selected editing can be exploited for gene dosage experiments [187] to evaluate the right combination in the desired phenotype without strong pleiotropic effects. This was observed, for example, when scientists attempted to modify the fatty acid composition of the camelina seed, especially when all the three homeologs gene copies of the Δ12-fatty acid desaturase (FAD2) genes were destroyed by the Cas9 activity [187,191]. Lee et. al’s study [191] shows that the triple knockout mutant plants significantly increased MUFA levels (up to 80%) in seeds, but led to a stunted bushy phenotype, and Mourineau et al. [187] confirmed a similar phenotype in T3 full-edited generations. No particular phenotype was described by Jiang et al. [186], who increased the oleic acid content from 16% to ≥50% in mutant seeds containing only homozygous or biallelic knock-outs of the FAD2 genes. This suggests that a reduction in PUFA within the vegetative tissues could impact agronomic performance, partly due to the expression of FAD2 in both seeds and vegetative tissues [193]. Aznar-Moreno [188] obtained an alteration in the seed oil composition by editing the three copies of two genes involved in the TAG synthesis for industrial use of the camelina oil, but the T2 generations show wrinkled and darkened seed compared to the wild-type. Ozseyhan’s group [189], on the other hand, by changing the target gene for Cas9 activity, was able to decrease the content of very long-chain fatty acids (VLCFAs) C20-C24 in seeds to 60% by inducing editing in all three fatty acid elongase 1 (FAE1) genes. In this case, no differences were observed between wild-type and edited seeds, suggesting that FAE1 genes do not adversely affect plant growth [189]. As shown in Table 2, genome editing applications in *C. sativa* are currently performed mainly to improve seed quality traits. In fact, the other two important traits that have been manipulated are the storage protein quality [190] and the amount of glucosinolates (GSLs) [133]. Lyzenga’s group improved the nutritional protein amino acid profile of camelina meal, editing only cruciferin C (*CsCRUC*) genes, the most divergent at the amino acid level and the most highly expressed among the 12 genes encoding cruciferin storage proteins. Consequently, seed amino acid content was positively changed with an increase in alanine, cysteine, and proline, and a decrease in isoleucine, tyrosine, and valine [190]. However, the knockout of the *CsCRU* genes did not show any significant alteration in total seed oil content, but increased the abundance of saturated fatty acids (SFAs). Whereas no effect on fatty acid (FA) composition, oil, and protein content was observed by Hölzl et al. [133] modulating the GSLs content in the seeds. Glucosinolates and their toxic breakdown products are the most undesirable antinutritional compounds [85,194], which limit the use of camelina seeds in animal feeding [41,195], but also act as plant defence compounds [196]. For this reason, by targeting the two major GSL transporters, GTR1, and GTR2, Hölzl et al. [133] obtained a reduction of 85–88% in GSL amount in the mutant seeds compared to the wild type. Furthermore, they also demonstrated that the editing of the transcription factors CsMYB28 and CsMYB29 resulted in the complete loss of GSLs in the seeds representing *C. sativa*, the first crop GSL-free of the *Brassicaceae* family. 

The cases discussed above suggest that CRISPR/Cas9 can be used to “knock out” or disable the target gene in competing pathways to direct metabolic flux toward the desired route. The choice and the selection of the desired ideotype, minimizing adverse effects, is possible by selecting the most efficient combination of the right target and the number of mutated alleles. The phenotype in this mutated background would also be more genetically stable than that achieved through RNAi, and better than a transgenic approach where transgenes are randomly integrated into the plant genome. Moreover, to date in the USA, edited plants that no longer contain exogenous DNA sequences from *Agrobacterium tumefaciens* T-DNA, but only contain the modification are not regulated by the US Department of Agriculture’s (USDA)-Animal and Plant Health Inspection Service’s (APHIS) as health-risk plants. In 2018, the Yeld10 Bioscience, an agricultural bioscience company, submitted to the Authorities a genome-edited camelina line in which all six copies of a gene involved in oil biosynthesis and oil turnover were single-based edited. In 2021, field evaluations in diverse geographical regions in the U.S. were conducted to evaluate the agronomic performances, confirming the genotype stability of the line, and a 5% increase in seed oil content as a percentage of seed weight over control plants [197]. 

In the EU, however, organisms developed with GE are not exempt from specific regulations that still determine which regulatory framework is warranted for these crops. Genetically modified crops and new breeding techniques are seen in Italy as a possible solution to the challenges posed by climate change. In June 2022, at the European Parliament, Italy’s Minister asked that the use of new plant breeding techniques should be liberalized by “untying them from GMO rules of the 1999 directive”. 

Currently, in the EU, plants’ characteristics resulting from GE applications could be protected by a patent where the origin of the mutation must be declared, even when the edit could also have occurred naturally [198]. 

However, in general, many methodologies could be used to improve *C. sativa* always keeping in mind that by intraspecific and inter-specific crosses, we can obtain new varieties. In contrast, by recurrent backcrosses, mutational breeding, transgenesis, and genome editing, we can obtain essentially derived varieties regulated by EU legislation [199].

## 7. Conclusions and Future Perspectives 

Although the breadth of research in camelina in the last few years is notable, several areas that would benefit from further research were identified. Winter-hardy oilseed cover crops show tremendous promise for providing needed ecosystem services. However, low seed yields and other quality traits currently hamper winter camelina’s potential to be economically viable for double or relay cropping, not only in the US Great Plains or upper Midwest, but also in other countries, such as Italy. Development of populations from crosses between winter and spring types of *C. sativa*, combined with leveraging next-generation sequencing technologies to identify genetic factors associated with, for example, freezing tolerance, flowering, yield, seed oil quantity and quality, are needed. Either through conventional breeding, transgenic approaches and RNAi and CRISPR/Cas 9 technology, increasing the seed size, seed yield, and oil quality in winter types of camelina could enhance their economic value as cover crops in double and relay cropping systems. Nevertheless, new research will be needed. In particular, new breeding programs and molecular genetics studies will allow us to achieve real use of this crop in our environment, promoting the rethinking of new, appropriate cropping systems, and moving into an era of climate-smart agriculture.

## Figures and Tables

**Table 1:** Uses of *Camelina sativa* (modified from Berti et al., 2016 [3]).

Uses	Details		References
Human nutrition	Food		[28,29,30,31,32,33]
	Diet supplements		
Animal feed	Bird	Chicken broilers	[19,34,35,36,37,38,39]
		Laying hens	
	Mammals	Cows	[40,41,42,43,44,45,46,47,47]
		Swine	
		Sheep	
		Rabbit	
		Swine	
	Fish	Salmon	[48,49,50,51,52,53,54,55,56]
		Trout	
		Other fish	
Chemicals	Polymers		[57,58,59,60,61,62,63,64]
	Adhesives		
	Resins		
	Cosmetics ingredients		
Fuels	Biodiesel		[1,3,5,13,64,65,66,67,68,69,70,71,72]
	Jet fuel		

**Table 2:** Select examples of camelina seed improvement performed by engineering techniques.

Seed Quality Traits Improved	Biotechnological Approach Used	Target/ Introduced Gene(s)	Promoter Used	Selectable Marker	Final Product/Major Results	Reference
Seed yield increase	single transgene overexpression	Arabidopsis purple acid phosphate (*AtPAP2*)	constitutive promoter not specified	BASTA (Bar gene) herbicide	50% higher seed yields with increased seed size	[160]
	single transgene overexpression	Arabidopsis G-protein γ subunit 3 (*AGG3*)	CaMV35S and seed-specific soybean glycinin	DsRed fluorescence and Bar gene	Increased seed size, number, and seed mass	[161]
	single transgene expression	Arabidopsis WRINKLED1 (AtWRI1)	Seed-specific SiW6 promoter	BASTA herbicide	Enhances seed oil content, seed mass and seed size	[162]
	transgenes cassette overexpression	E.coli chloroplast glycolate dehydrogenase (GDH), glyoxylate carboxylase (GCL), and tartronic semialdehyde reductase (TSR)	CaMV35S promoter, tobacco EntCUP4 promoter, Arabidopsis ACTIN2 promoter	seed mCherry fluorescence, phosphinothricin herbicide	enhanced CO2 use efficiency increased plant grown up to 50%	[163]
	transgenes cassette expression	Arabidopsis diacylglycerol acyltransferase1 (DGAT1), and a yeast cytosolic glycerol-3-phosphate dehydrogenase (GPD1)	seed specific oleosin and glycinin promoters from soybean	DsRed fluorescence and Bar gene	up to 52% increase in seed mass, and up to 13% higher seed oil content	[164]
	single transgene expression	nonspecific phospholipase C6 (NPC6)	not specified	hygromycin B antibiotics	increase seed oil content, seed weight, and oil yield	[165]
	single transgene expression	chimeric arabidopsis myosin XI-2	Arabidopsis myosin XI-2 promoter	hygromycin B antibiotics	improve plant growth, total seed yield increase as the total seed number	[166]
	transgenes cassette overexpression	At lipid transporters, FAX1 (fatty acid export1), and ABCA9 (ATP-binding cassette transporter subfamily A9)	CaMV35S promoter	kanamycin antibiotic in plates	increased expression of fatty acid, and seed oil production, increased seed weight and size	[167]
Seed protein content	RNAi suppression	12S and 2Sinapin protein	seed specific soybean glycine 1	DsRed fluorescence and Bar gene	seed storage protein (SPP) modulation	[168]
	RNAi suppression	ADP-glucose pyrophosphorylase (AGPase)	seed-specific phaseolin promoter	DsRed fluorescence	enhanced seed protein content and seed size	[169]
Seed oil modulation	RNAi suppression	camelina FAD2 and FAE1	not specificized	DsRed fluorescence and Bar gene	increase up to 50% oleic acid	[168]
	RNAi suppression	fatty acid desaturase 3 (FAD3) and FAE1	soybean glycinin-1 promoter	DsRed fluorescence and Bar gene	seeds with high linoleate content (approximately 57% of total FA)	[170]
	transgenes cassette expression	set of genes of Δ6-desaturase pathway	Different seed-specific promoters, such as Arabidopsis FAE1 promoter, flax Cnl1 and Cnl2, and *Brassica napus* napin promoter	BASTA herbicide	>12% of DHA, high ω3/ω6 ratio	[170,171]
	transgenes cassette expression	microalgal and yeast set of genes for EPA synthesis	Different seed-specific promoters, such as *Vicia faba* USP, and sucrose binding protein promoter; napin promoter, flax seed specific conlinin 1 (Cnl1)	DsRed fluorescence protein	EPA and DHA content levels in camelina equivalent to those in fish oils	[49]
	transgenes cassette expression	*Ricinus communis* fatty acid hydroxylase (RcFAH), and Lesquerella condensing enzyme gene (LfKCS3)	native promoter of camelina and seed-specific phaseolin promoter	BASTA herbicide	high levels of hydroxyl fatty acid	[172]
	single transgene and transgenic cassette expression	*Lunaria annua* Ketoacyl-CoA synthase (KCS) and the other three elongase genes from Arabidopsis	seed specific soybean glycin1 and oleosin1, cassava vein mosaic virus (CMVP) promoter	DsRed fluorescence protein	higher VLCFA production, in particular of 6-12% (C24:1Δ15) nervonic acid	[173]
	transgenes cassette expression	eight different acyl-carrier-thioesterase (FATB) from *Caesalpinia pulcherrima*, *Cuphea viscosissima*, *Crocodylus palustris*, *Cladopus hookeriana* and *Umbellularia californica*	soybean glycinin-1 promoter	DsRed fluorescence protein	medium chain FA of different lengths accumulation	[60]
	single transgene overexpression	Arabidopsis patatin-related phospholipase *pPLAIIIδ*	35S promoter, soybean glycinin1 promoter	hygromycin B antibiotics	Increased seed oil content and decreasing cellulose content	[58]
	single transgene expression and RNAi	*Euonymus alatus* diacylglycerol-acetyltransferase (DAcT) overexpression with suppression of DGAT1 and/or PDAT1	seed specific soybean glycin1 and oleosin1	DsRed fluorescence protein	modification and increased level of triacylglycerol content, seed yield improvement	[174]
	camelina gene overexpression	camelina DGAT1B	Seed-specific *Brassica napus* Napin promoter	BASTA herbicide	Total seed oils were increased by ~24%	[175]
	single transgene expression and RNAi	*Umbellularia californica* 12-acyl-carrier thioesterase (FATB) expression and KASII suppression	seed specific napin promoter	mCherry fluorescence gene	higher accumulation up to 28.5% of palmitate, reduction in longer, unsaturated fatty acids in seed TAGs.	[176]
	overexpression and down-regulation using artificial microRNA (amiRNA)	PDAT overexpression and DGAT suppression	seed specific napin promoter	BASTA herbicide	oil modulation: a-linolenic decrease and linoleic acid increase	[177]

**Table 3:** Applications of CRISPR/Cas9 editing technology in camelina.

Cultivar	Type/Promoter for Cas9	Promoter for gRNA Expression	Target Genes	sgRNA Features	Selection Marker	Mutant line Detection System	Trait/Phenotype	References
Suneson	Constitutive/ 35S promoter from the Cauliflower mosaic virus (CaMV35S)	*Arabidopsis thaliana* U6 promoter (AtU6-26)	fatty acid desaturase 2 (FAD2) genes	three independent sgRNAs on a conserved region of the 3 FAD2 genes, all designed in 5′-3′ (forward) direction	Red fluorescent protein (DsRed)	restriction enzyme screening	increased MUFA (monosaturated fatty acid) content in the seed	[186]
Celine	Constitutive/ Ubiquitin 4−2 promoter from Petroselinum crispum (PcUbi4-2)	*Camelina sativa* U6 promoter (CsU6)	fatty acid desaturase 2 (FAD2) genes	Two independent sgRNAs on a conserved region in the first 600bp of the 3 FAD2 genes, one in 5′-3′ direction, one in 3′-5′ direction	DsRed	simple allele-discriminating PCR (SAP)	increased MUFA (monosaturated fatty acid) content in the seed	[187]
Suneson	Constitutive/ CaMV35S	AtU6-26	phospholipid: diacylglycerol acyltransferase 1 (PDAT1), diacylglycerol acyltransferase (DGAT1) genes	One sgRNA on a conserved region of the three PDAT1 genes and 1sgRNA for the 3DGAT1. Both sgRNA are designed in 5′-3′ direction	Hygromycin phosphotransferase	restriction enzyme screening	Reduced oil content and altered fatty acid composition	[188]
Suneson	Tissue-specific/ Egg cell-specific promoter (EC1.1)	AtU6-26	fatty acid elongase 1 (FAE1) genes	One sgRNA in reverse strand (3′-5′) in a conserved region in the first 600bp of the three FAE1 genes	DsRed	sequencing of the target regions	Decreased VLCFAs C20-C24 from 22% to 2%	[189]
Doubled haploid line DH55	Constitutive/Arabidopsis thaliana Elongation factor 1 (AtEF1a)	AtU6-26	CRUCIFERIN C (CRUC) genes	2sgRNAs in 5′-3′ and 3′-5′ direction, respectively on the first 600bp of the three target genes	Glufosinate-ammonium	droplet digital PCR (ddPCR)	increase in high value-amino acids proteins in the seed	[190]
Suneson, CAME	Tissue-specific/ Egg cell-specific promoter (EC1.1)	AtU6-26	fatty acid desaturase 2 (FAD2) genes	One single sgRNA in the first 300bp in a conserved region of the three FAD2 genes designed in 5′-3′ direction	DsRed	deep sequencing of the targeted sites	increased MUFA (monounsaturated fatty acid) content in the seed	[191]
CAM139	Constitutive/ PcUbi4-2	AtU6-26	glucosinolate transporter 1 and 2 (GTR1-GTR2); transcription factors MYB28, MYB29	Two sgRNAs in conserved regions for each of the three homeolog genes target (7 sgRNA in total) 1sgRNA in common between GTR1 and GTR2 and 1sgRNA in common between MYB28 and MYB29 designed in both directions	DsRed	restriction enzymes screening	decrease glucosinolate content in the seed	[133]
